# Deficits in high- (>60 Hz) gamma-band oscillations during visual processing in schizophrenia

**DOI:** 10.3389/fnhum.2013.00088

**Published:** 2013-03-26

**Authors:** Christine Grützner, Michael Wibral, Limin Sun, Davide Rivolta, Wolf Singer, Konrad Maurer, Peter J. Uhlhaas

**Affiliations:** ^1^Department of Neurophysiology, Max-Planck Institute for Brain ResearchFrankfurt am Main, Germany; ^2^MEG Unit, Brain Imaging Center, Johann Wolfgang Goethe UniversityFrankfurt am Main, Germany; ^3^Department of Radiology, Massachusetts General Hospital, Harvard University, CambridgeMA, USA; ^4^Ernst-Strüngmann Institute for Neuroscience in Cooperation with Max-Planck SocietyFrankfurt am Main, Germany; ^5^Frankfurt Institute for Advanced Studies, Johann Wolfgang Goethe UniversityFrankfurt am Main, Germany; ^6^Department of Psychiatry, Johann Wolfgang Goethe UniversityFrankfurt am Main, Germany; ^7^Institute of Neuroscience and Psychology, University of GlasgowGlasgow, UK

**Keywords:** MEG, gamma, schizophrenia, perceptual organization, synchrony

## Abstract

Current theories of the pathophysiology of schizophrenia have focused on abnormal temporal coordination of neural activity. Oscillations in the gamma-band range (>25 Hz) are of particular interest as they establish synchronization with great precision in local cortical networks. However, the contribution of high gamma (>60 Hz) oscillations toward the pathophysiology is less established. To address this issue, we recorded magnetoencephalographic (MEG) data from 16 medicated patients with chronic schizophrenia and 16 controls during the perception of Mooney faces. MEG data were analysed in the 25–150 Hz frequency range. Patients showed elevated reaction times and reduced detection rates during the perception of upright Mooney faces while responses to inverted stimuli were intact. Impaired processing of Mooney faces in schizophrenia patients was accompanied by a pronounced reduction in spectral power between 60–120 Hz (effect size: *d* = 1.26) which was correlated with disorganized symptoms (*r* = −0.72). Our findings demonstrate that deficits in high gamma-band oscillations as measured by MEG are a sensitive marker for aberrant cortical functioning in schizophrenia, suggesting an important aspect of the pathophysiology of the disorder.

## Introduction

Abnormal changes in neural oscillations have received widespread attention in recent years as an important mechanism for understanding the pathophysiology of schizophrenia (Uhlhaas and Singer, 2010). This is due to their fundamental role in establishing precise temporal relationships between neural responses at different spatial scales within and across cortical regions. Oscillations in the beta/gamma range establish synchronization with great precision in local cortical networks (Gray et al., 1989; Womelsdorf et al., 2007). Neural oscillations are a functionally relevant phenomenon as they enhance signal transmission (Fries et al., 2007), modulate synaptic plasticity (Wespatat et al., 2004), and correlate with a range of cognitive functions, including basic sensory processes (Gray et al., 1989), executive functions (Roux et al., 2012), and consciousness (Melloni et al., 2007) [for a recent review see Uhlhaas et al. (2009)].

Schizophrenia is associated with impairments in all these domains as well as psychotic symptoms that involve disturbance in conscious experience. These deficits may involve a disconnection syndrome within and between brain regions (Andreasen, 1999; Phillips and Silverstein, 2003; Stephan et al., 2009) that underlies dysfunctional coordination in local and extended cortical circuits. Accordingly, abnormal oscillations may parsimoniously explain core features of schizophrenia. In addition, they can be mapped onto distinct neural processes as much is known about the physiological and anatomical mechanisms underlying the generation of synchronized neural oscillations. The network of GABAergic interneurons has pace-maker functions for the generation of gamma-rhythms in local cortical circuits (Sohal et al., 2009; Oke et al., 2010), while the excitatory cortico-cortical connections are essential for long-range synchronization in the beta- and gamma-band (Engel et al., 1991).

So far most studies focused on abnormal oscillations in the 40–70 Hz frequency range as a putative cause for the cognitive deficits in schizophrenia (Kwon et al., 1999; Spencer et al., 2003; Cho et al., 2006) because oscillations around ~40 Hz were initially proposed to serve as a mechanism for the binding of spatially distributed responses in vision (Gray et al., 1989). These studies revealed robust abnormalities in auditory steady state potentials to 40 Hz stimulation (Kwon et al., 1999) and consistent evidence for deficits in phase-synchrony of evoked and induced oscillations in the gamma frequency range (Spencer et al., 2003; Uhlhaas et al., 2006a). In contrast, reductions in the amplitude of induced and evoked gamma-band oscillations during sensory processing and higher cognitive functions have been conflicting, with some studies showing circumscribed impairments (Cho et al., 2006) while other studies could not confirm these findings (Uhlhaas et al., 2006a).

Reasons for the inconsistent findings in regards to reductions in gamma-band spectral power could be the focus on oscillatory activity in the low (25–60 Hz) gamma frequency range. More recent evidence from magnetoencephalographic (MEG) studies (Guggisberg et al., 2007; Siegel et al., 2007; Chaumon et al., 2009; Grutzner et al., 2010), invasive recordings in monkeys (Jutras et al., 2009), and intracranial electroencephalographic (iEEG) recordings in humans (Lachaux et al., 2005; Crone et al., 2006) suggest, however, that gamma-band oscillations extend to activity up to 200 Hz.

This so-called “high” gamma-band activity (60–200 Hz) may be important for cortical computations and can be measured with a high signal-to-noise ratio (Hoogenboom et al., 2006). Indeed, there is a good agreement between the high gamma-band activity disclosed by MEG and oscillatory activity measured by iEEG (Dalal et al., 2009), suggesting a close correspondence between non-invasively recorded high gamma-band oscillations through MEG and directly recorded neural activity.

The contribution of high gamma-band activity toward the pathophysiology of schizophrenia is, however, unclear. A recent EEG study provided preliminary evidence for a circumscribed deficit in high gamma-band activity during the delay period of a working memory task in schizophrenia patients (Haenschel et al., 2009). Similarly, Hamm et al. (2011) showed that deficits in auditory steady-state responses (ASSRs) also involve deficits to entrainment at 80 Hz.

In the current experiment, we employed MEG to examine comprehensively the role of low and high gamma-band activity. Moreover, there is an important advantage of MEG over EEG in the measurement of high-frequency oscillations in that the magnetic field can be measured undisturbed by tissue inhomogeneities. This results in a superior signal-to-noise (SNR) in MEG- relative to surface EEG-recordings (Muthukumaraswamy and Singh, 2013) as well as improved localization accuracy for the generators of cortical high frequency oscillations (Kaiser and Lutzenberger, 2005).

As in our previous study with EEG (Uhlhaas et al., 2006a), Mooney faces as a test of perceptual organization were used. A large body of research suggests that in addition to dysfunctions in higher cognitive functions, such as executive processes and working memory, schizophrenia is also associated with deficits in perceptual processing (Uhlhaas and Silverstein, 2005; Uhlhaas and Mishara, 2007). This is supported by psychophysical (Uhlhaas et al., 2006b; Javitt, 2009), anatomical (Selemon et al., 1995), and physiological data (Krishnan et al., 2005) that indicate abnormalities in sensory functions.

## Materials and methods

### Participants

Sixteen patients with schizophrenia were recruited from in- and out-patient units from the Frankfurt University Psychiatry Department. Sixteen healthy controls were recruited from the local community and screened for psychopathology with the German version of Structured Clinical Interview for DSM-IV-R Non-Patient Edition (SCID) (Saß et al., 2003). Written informed consent was obtained from all participants following a description of the study procedures. The study was carried out according to the Declaration of Helsinki and approved by the ethical committee of the Johann Wolfgang Goethe-University Frankfurt. DSM-IV diagnosis of schizophrenia was established with the SCID-Interview, by thorough chart review and in consultation with the treating psychiatrists. Patients and controls were excluded if they had any neurological or ophthalmologic disorders that might have affected performance or if they met criteria for alcohol or substance dependence within the last month.

All patients were on medication at the time of testing with 15 out of 16 receiving atypical antipsychotic medication. Six patients with schizophrenia were treated with a combinatory therapy of different antipsychotics. Moreover, two patients received a mood stabilizer.

Current psychopathology was assessed with the Positive and Negative Syndrome Scale (PANSS) (Kay et al., 1987) and symptoms were grouped into five factors according to the model of Lindenmayer et al. (1995), including the factors “positive,” “negative,” “depression,” “excitement,” and “cognitive.” In addition, we rated schizophrenia patients on the item “inappropriate affect” (Cuesta and Peralta, 1995) to allow for an assessment of the factor “disorganization” which comprises the items conceptual disorganization, poor attention, and inappropriate affect.

Demographic information for patients and controls is given in Table 1. Patients with schizophrenia and healthy controls were of similar age and education. No differences between groups were found for premorbid verbal IQ and handedness. Cognitive function in patients and controls was measured with the Brief Assessment of Cognition in Schizophrenia (Keefe et al., 2004). Compared to healthy controls, schizophrenia patients had lower scores on all scales of the BACS (verbal memory, digit sequencing, motor speed, verbal fluency, and symbol coding) except for the tower of London test (Table 1).

**Table 1:** **Means, standard deviations, and mean differences for demographic, neurocognitive, and clinical characteristics of controls and schizophrenia patients**.

	**Healthy controls** (***N*** = **16**)		**Chronic patients** (***N*** = **16**)		**Statistics**	
	**Mean**	***SD***	**Mean**	***SD***	***X*^2^-/*t*-value**	***p*-value**
Gender (m/f)	8/8		8/8		*X*^2^_(1)_ = 0.52	0.47
Age (years)	34.19	10.56	38.25	9.38	*t*_(30)_ = −1.15	0.26
Education (years)	15.33	3.11	14.43	2.95	*t*_(27)_ = 0.80	0.43
Handedness	69.22	31.45	71.80	25.35	*t*_(29)_ = −0.25	0.80
MWT	30.67	2.85	28.47	3.16	*t*_(28)_ = 2.00	0.05
**BACS**						
Verbal memory	52.00	7.51	36.13	13.22	*t*_(28)_ = 4.04	0.0004
Digit	25.13	3.96	20.13	4.12	*t*_(28)_ = 3.39	0.0021
Motor	88.80	9.28	75.73	11.44	*t*_(28)_ = 3.43	0.0019
Fluency	58.67	14.05	40.47	9.93	*t*_(28)_ = 4.10	0.0003
Symbol cod.	57.21	10.24	45.93	16.38	*t*_(27)_ = 2.20	0.036
ToL	19.73	2.28	18.33	2.82	*t*_(28)_ = 1.49	0.15
Total score	298.47	31.58	236.73	41.95	*t*_(28)_ = 4.55	0.0001
**PANSS**						
Negative	–	–	18.13	5.60		
Excitement	–	–	6.31	1.82		
Cognitive	–	–	10.25	3.45		
Positive	–	–	9.25	3.59		
Depression	–	–	12.75	3.47		
Disorganization	–	–	5.69	2.44		
Total score			69	17.04		

### Stimuli and task

**Figure 1:**
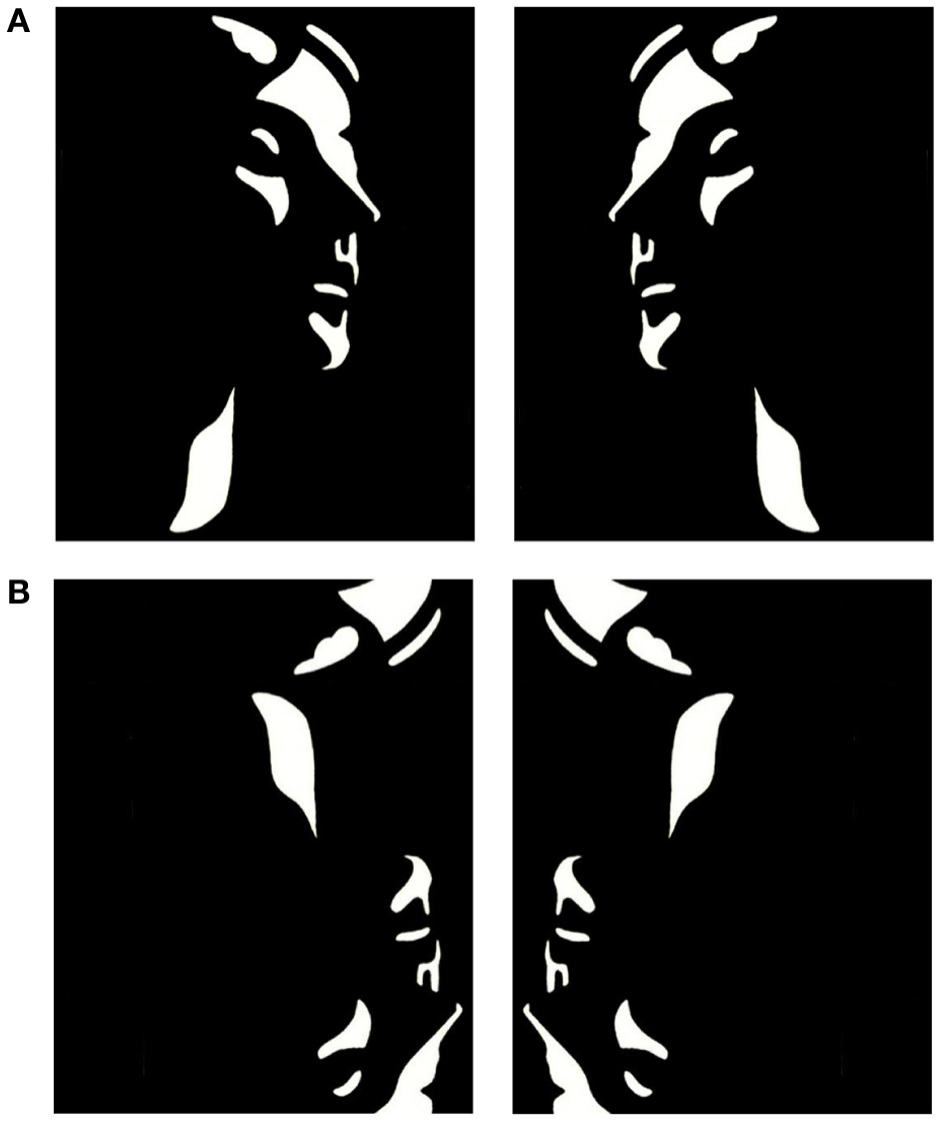
**Examples of upright (A) and inverted-scrambled (B) Mooney face stimuli**.

Mooney and Ferguson (1951) developed a visual closure task consisting of degraded pictures of human faces where all shades of gray are removed, thereby leaving the shadows rendered in black and the highlights in white. Perception of Mooney faces involves the grouping of the fragmentary parts into coherent images based on the Gestalt principle of closure. We used a set of 160 different stimuli, consisting of the 40 original Mooney stimuli presented in the upright orientation, mirrored at the vertical axis and in corresponding versions mirrored at the horizontal axis (Figure 1). To decrease the likelihood of perceiving a face in inverted stimuli, images were distorted through either slightly rearranging the configuration of white or black patches or changing the contours of black or white background areas. This distortion ensured that no faces were perceived in the inverted condition while minimally affecting low-level stimulus properties, such as luminance and spatial frequencies.

Participants were presented with a random sequence of upright and inverted-scrambled stimuli which were shown for 200 ms. The inter-stimulus interval ranged between 3500 and 4500 ms. Participants responded with a button press to both face and no-face stimuli and the hand assignment was counterbalanced across participants. A fixation cross was presented in the center of the screen between trials. Prior to data collection, participants performed a practice block to become familiar with the task and the response buttons. All participants completed four experimental runs, each of which was composed of 60 upright and 30 inverted-scrambled stimuli.

The stimuli were displayed in the center of a translucent screen at a viewing distance of 53 cm and subtended 19° of visual angle. An LCD projector located outside the magnetically shielded room of the MEG was used to project the stimuli onto the screen via two front-silvered mirrors. Stimulus presentation was controlled using the Presentation software package (Neurobehavioral Systems, Inc.).

### MEG data acquisition

MEG data were recorded continuously using a 275-channel whole-head system (Omega 2005, VSM MedTech Ltd., BC, Canada) at a rate of 600 Hz in a synthetic third order axial gradiometer configuration (Data Acquisition Software Version 5.4.0, VSM MedTech Ltd., BC, Canada). The data were filtered with 4th order butterworth filters with 0.5 Hz high-pass and 150 Hz low-pass. Behavioral responses were recorded using a fiberoptic response pad (Lumitouch, Photon Control Inc., Burnaby, BC, Canada) on the stimulus PC and fed through to the MEG acquisition system as an additional channel. Before and after each run, the subject's head position relative to the gradiometer array was measured using coils placed at the subject's nasion, and 1 cm anterior to the tragus of the left and right ear. Runs with a head movement exceeding 5 mm were discarded.

### MEG data processing

MEG data were analyzed using the Fieldtrip open source Matlab toolbox (http://www.ru.nl/fcdonders/fieldtrip/). Trials were defined from the continuously recorded MEG from −1000 ms to 1000 ms with respect to the onset of the visual stimulus and classified according to the two experimental conditions, the face condition, containing trials with upright stimuli, and the non-face condition, containing trials with inverted-scrambled stimuli. Only data with correct responses were considered for all further analyses.

Data epochs contaminated by eye blinks, muscle activity, or jump artifacts in the SQUIDs were discarded using automatic artifact detection and rejection routines provided by the Fieldtrip software. Non-artifact trials were baseline-corrected by subtracting the mean amplitude during an epoch ranging from −500 to −100 ms before stimulus onset.

### Analysis of spectral power changes

Time-frequency representations (TFRs) were computed by means of Morlet wavelets with a width of 5 cycles per wavelet at center frequencies between 25 and 140 Hz, in 1 Hz steps.

Task-related differences in spectral-power were analyzed in two steps. Firstly, we compared spectral power within each group in the face condition (50–350 ms post-stimulus) to the baseline raw power. In addition to estimating power-values, we also computed inter-trial phase-coherence (ITPC) (Delorme and Makeig, 2004) across all sensor-groups. This analysis approach was used to distinguish transient oscillatory activity associated with the on- and off-responses from induced, non-phase locked oscillations based on the gradient of ITPC-values. Statistical results were corrected for multiple comparisons in space, time, and frequency with false discovery rate (FDR) (Genovese et al., 2002) with a criterion of *q* < 0.05.

In a second step, we calculated the contrasts (*t*-tests) of a non-parametric 2 × 2 ANOVA based on a permutation of residuals approach (Anderson and Ter Braak, 2003), with factors group (controls vs. ScZ) and condition (face vs. no-face) for the evoked and induced time windows. We ran 1500 permutations for each test aimed at investigating the main effects and the interaction of the ANOVA design. Statistical results were corrected for multiple comparisons in space, time, and frequency means of a cluster-based correction (Maris et al., 2007).

### Psychophathology and gamma-band power

In order to compute correlations between gamma-band power, clinical symptoms, and performance, we averaged absolute task-related power in the low and high gamma-band ranges in the face condition for each patient and control participant over the significant channels of the respective group. To determine significant channels, the permutation *t*-values given by the statistical analysis were first multiplied with the significance mask that contained zeros for all non-significant time-frequency-channel samples, and ones for all significant samples; this way we obtained a matrix where all *t*-values unequal 0 were statistically significant. This matrix was then averaged across the time and frequency range used in the statistical analysis (50–350 ms and 25–140 Hz). To determine the channels showing on average a significant increase (further on denoted as “positive” channels) or decrease (“negative” channels) in power, we set a threshold of *t* > 0 (positive channels) and *t* < 0 (negative channels).

### Effect-sizes for sensor-level spectral power changes

We computed effect sizes for differences between the control and patient groups for both positive and negative clusters between 50 and 350 ms in the 25–140 Hz frequency range in the face condition. We averaged absolute power values between the respective frequency bands over positive and negative cluster channels separately for controls and schizophrenia patients. Effect sizes were then calculated by dividing the difference between the average gamma-band power in patients and controls by the pooled variance.

## Results

### Behavioral performance

We analysed the percentage of correct responses as well as reaction times for the face and the non-face condition (Table 2). Furthermore, we computed the discrimination index A′ = 0.5 + [(H-FA) × (1 + H-FA)]/[4 × H × (1-FA)] (H: Hits; FA: False Alarms) (Grier, 1971). A′ is a non-parametric measure of signal detection sensitivity that is based on both hits and false alarms (FA, inverted-scrambled stimuli classified as faces). Chronic schizophrenia patients detected significantly fewer faces than controls [*t*_(30)_ = 2.07, *p* = 0.047] and had longer reaction times [*t*_(30)_ = −2.07, *p* = 0.01]. No differences were found for behavioral performance in the non-face condition [correct responses: *t*_(30)_ = −1.06, *p* = 0.3; reaction times: *t*_(30)_ = 0.48, *p* = 0.64]. The significant difference between groups in A′ [*t*_(30)_ = 2.09, *p* = 0.046] confirmed that controls had a better discrimination performance compared to patients.

**Table 2:** **Means, standard deviations and mean differences for behavioral performance in controls and patients**.

	**Healthy controls (*N* = 16)**		**Chronic patients (*N* = 16)**		**Mean difference**	
	**Mean**	***SD***	**Mean**	***SD***	***t*-value**	***p*-value**
Hits (%)	80.59	6.67	74.03	10.78	*t*_(30)_ = 2.07	0.047
Correct rejections (CR) (%)	86.59	11.09	84.87	9.33	*t*_(30)_ = 0.48	0.64
Discrimination index A′	0.91	0.04	0.88	0.03	*t*_(30)_ = 2.09	0.046
Reaction time (hits) (ms)	610.04	78.30	688.19	85.45	*t*_(30)_ = −2.70	0.01
Reaction time (CR) (ms)	754.38	86.40	792.08	112.23	*t*_(30)_ = −1.06	0.30

### ITPC-analysis

**Figure 2:**
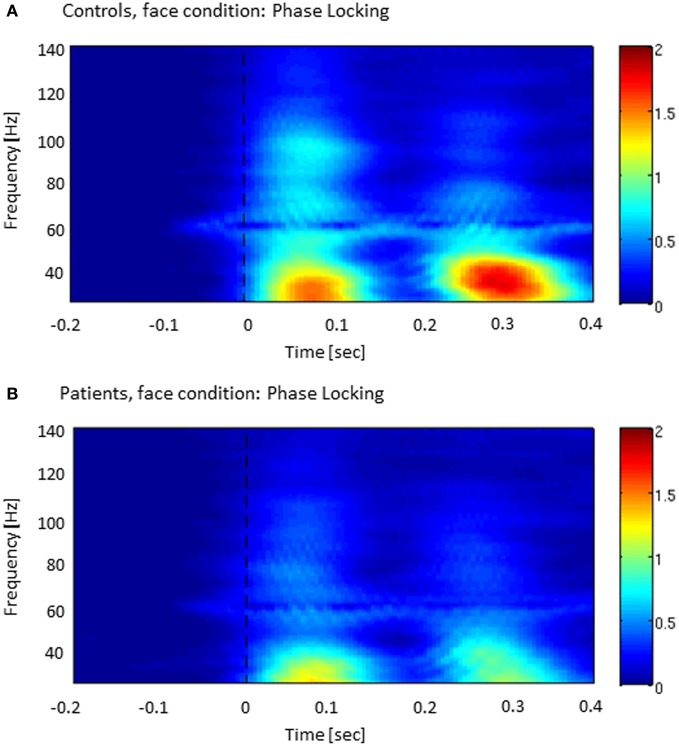
**Inter-trial phase-coherence (ITPC) across all sensor-groups in both controls (A) and patients with schizophrenia (B).** The colored scale (0–2) indicates change in ITPC relative to baseline.

The analysis of ITPC-values revealed prominent increases in the low gamma-band range during an early (5–120 ms) and a later time window (220–320 ms) (Figure 2), which likely reflected transient activity related to the on and offset response of the stimulus. Accordingly, we defined three time windows: (1) an early evoked time window (onset-response: 5–105 ms); (2) an induced period (105–220 ms); and (3) a second evoked window (offset-response: 220–320 ms).

### Gamma-band power at sensor level

**Figure 3:**
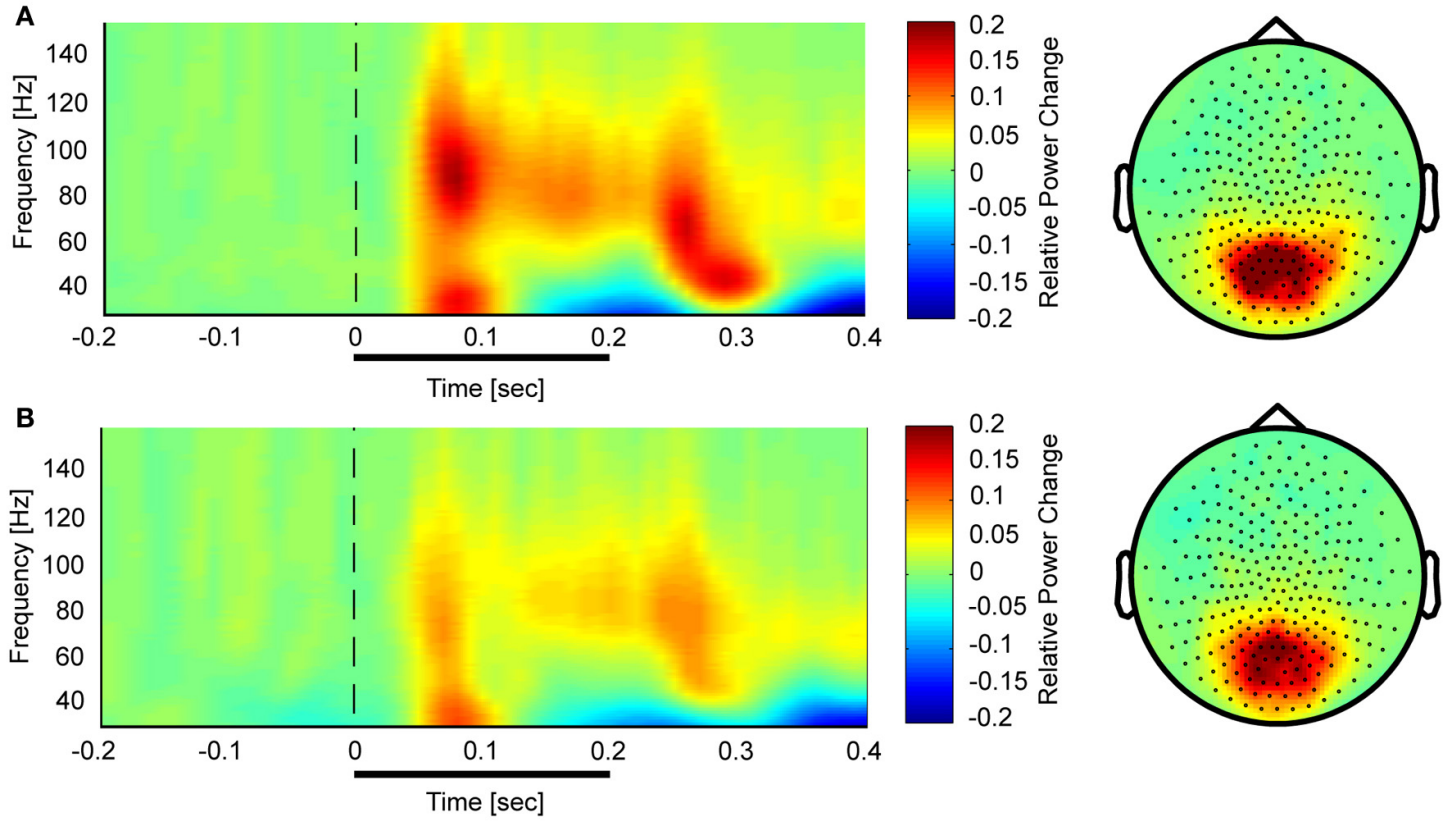
**Time-frequency representations and topographies of gamma-band spectral power in the face condition for controls (A) and schizophrenia patients (B).** The gamma-band signal is expressed as relative power change in the post-stimulus time window compared to baseline, averaged across all channels. The topographies are averaged across the post-stimulus interval (0–400 ms) and between 25 and 150 Hz.

Controls showed a task-related increase between 50 and 350 ms after stimulus onset with two prominent gamma-band peaks around 50 and 250 ms in the 25–140 Hz frequency (Figure 3). We observed sustained gamma-band activity between 100 and 300 ms, mainly between 60 and 120 Hz.

**Figure 4:**
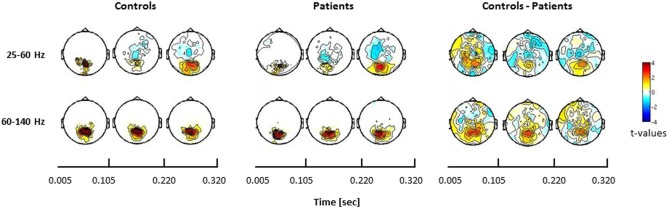
**Statistical analysis of power changes in response to upright Mooney faces for controls (left panel), schizophrenia patients (middle panel), and for the difference between controls and patients (right panel) for three time windows (onset-response, induced period, offset-response).** The topographies for controls and schizophrenia patients show significant differences between the face condition and the 0.5 s pre-stimulus baseline, separately for the lower (25–60 Hz) and the higher (60–140 Hz) gamma-band. Red denotes higher activation during stimulus presentation compared to baseline, whereas blue denotes less activation during stimulus presentation compared to baseline. Right panels show the difference for the face condition between controls and patients. Here, red denotes stronger activation for controls compared to patients, whereas blue represents stronger activation in patients relative to controls. The effects are masked by the significance map derived from false-discovery rate (FDR, *q* < 0.05) statistical testing.

In the face condition, post-stimulus activity in controls revealed a significant increase in low- and high-frequency gamma-band power over parieto-occipital channels between 50 and 350 ms. In schizophrenia patients, relative gamma-band power averaged across all channels was characterized by strongly reduced low and high gamma-band power compared to controls for both evoked and induced time-windows (Figure 4). The reduction of gamma-band power was especially pronounced in the high gamma-band range (60–140 Hz) (effect size: *d* = 1.26) while the deficit in the lower gamma-band was considerably smaller (effect size: *d* = 0.71). In addition, there was a significantly stronger gamma-band activity in the 25–60 Hz range over fronto-central channels in schizophrenia patients relative to controls. This relative increase was due to a reduced downregulation of gamma-band power over fronto-central channels in patients since gamma-band power was decreased over fronto-central channels in controls (*d* = −0.31).

**Figure 5:**
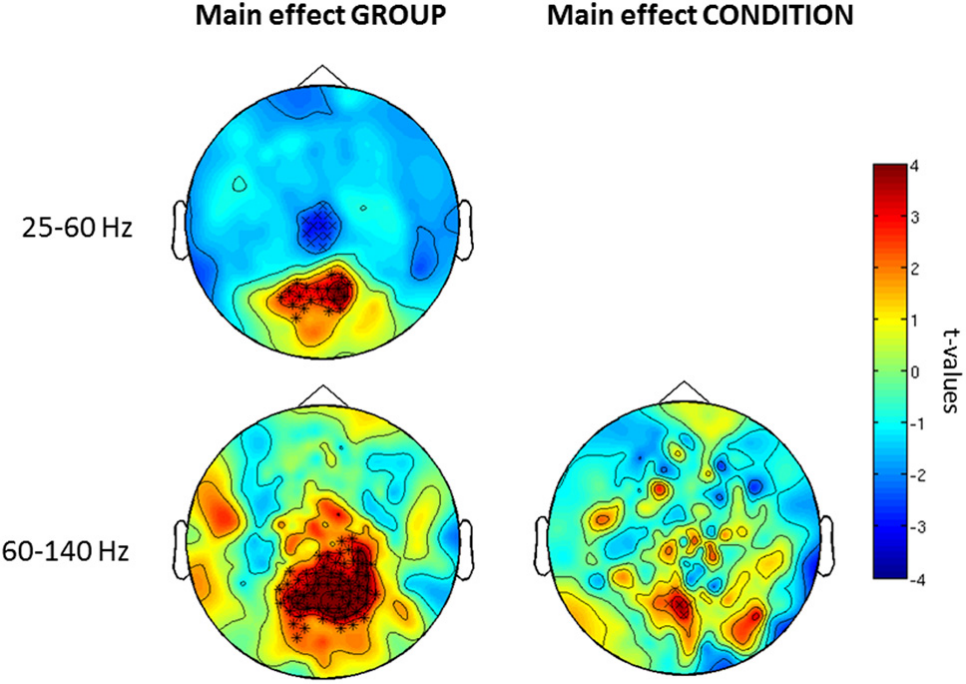
***Post-hoc* contrasts for the non-Parametric ANOVA indicating the main effects of group (left) and condition (right) for both low (top) and high (bottom) gamma-band oscillations at the sensor level.** For the main effect of group (left column), red colors indicate increased activity in controls while blue color suggests increased gamma-band power in schizophrenia patients relative to controls. For the main effect of condition (right column), red colors indicate higher gamma-band activity to faces while increased spectral power to no-face stimuli is represented in blue. The topographies depict corrected *t*-values and the channels that form a statistically significant cluster are indicated (^*^, *p* < 0.001; x, *p* < 0.05).

Confirming these results, the 2 × 2 ANOVA revealed a main effect of group (Figure 5) at both high and low gamma-band frequencies and a main effect of condition in the high gamma-band over parieto-occipital sensors, suggesting an upregulation of 60–140 Hz activity during perceptual organization of Mooney faces which is consistent with prior findings from our group (Grutzner et al., 2010). There was, however, no statistically significant effect for the interaction between group and condition.

**Figure 6:**
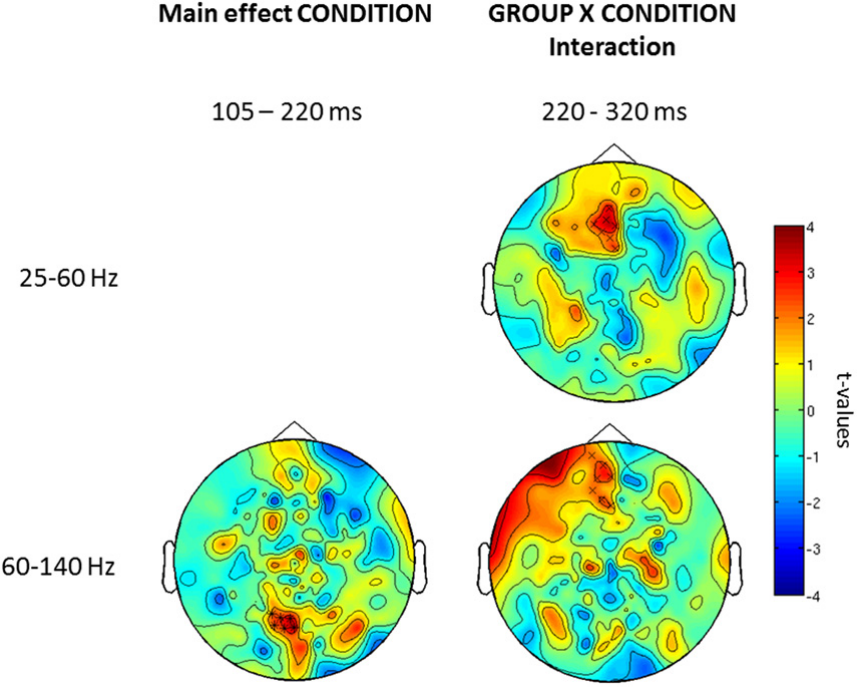
**Topography for the effect of condition (left) and group × condition (right) interaction for low and high gamma-band oscillations for three time windows (onset-response, induced period, offset-response).** Red clusters indicate higher gamma-band power in the face vs. the no-face condition. The topographies depict corrected *t*-values and the sensors that form a statistically significant cluster are indicated (^*^, *p* < 0.001; *x*, *p* < 0.05).

Further examination of condition and group × interaction effects in induced vs. evoked time windows showed that the effect of condition was only found in the 105–220 ms window. Finally, we did find an interaction between group × condition for the offset response over frontal sensors (Figure 6).

### Baseline-analysis

To exclude that effects were solely driven by differences in baseline-activity, we also examined differences in baseline activity in the face condition prior to stimulus onset which could potentially bias differences task-related activity. Comparisons between schizophrenia patients and controls showed no differences in baseline power-values between groups.

### Correlations between clinical symptoms, behavior and gamma-band power

Correlations between clinical symptoms, behavior and gamma-band power in the face condition were computed separately with task-related absolute difference power on positive channels in the high gamma-band, with task-related absolute difference power on positive channels in the lower gamma-band, and finally with task-related absolute difference power on negative channels in the lower gamma-band.

**Figure 7:**
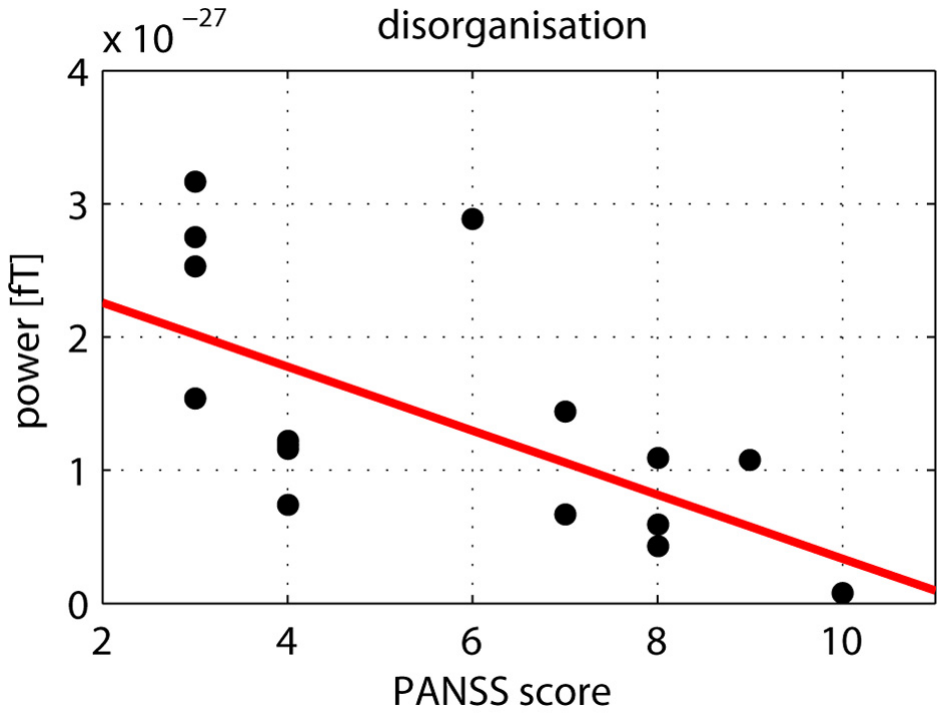
**Correlation between high gamma-band power and disorganization.** The scatter-plot shows the relationship between high (60–120 Hz) gamma-band power in the 50–350 ms time window over positive channels and the disorganization component of the PANSS.

After correcting for multiple comparisons for each change in gamma-band power with the six factors of the PANSS (alpha-level: *p* = 0.01), we found a significant correlation between reductions in high gamma-band power over positive channels and increased scores on the disorganization factor (*r* = −0.723, *p* = 0.002) (Figure 7). This relationship was also found for the reduction in low gamma-band activity over parietal channels (disorganization: *r* = −0.68, *p* = 0.006), while no other correlations reached the alpha-level of *p* = 0.01 (Table 3). The correlation between high gamma-band power and the discrimination index A′ reached the *p* = 0.05 level in schizophrenia patients but was not statistically significant after correcting for multiple comparisons (Table 4).

**Table 3:** **Correlations between gamma-band power and clinical symptoms**.

**PANSS**	**Healthy controls**		**Chronic patients**	
	**rho**	***p*-value**	**rho**	***p*-value**
	High gamma, Positive cluster			
Disorganization	–	–	−0.729	0.002
Depression	–	–	−0.133	0.635
Positive	–	–	−0.207	0.460
Cognitive	–	–	−0.555	0.032
Excitement	–	–	−0.435	0.105
Negative	–	–	−0.317	0.249
	Low gamma, positive cluster			
Disorganization	–	–	−0.677	0.006
Depression	–	–	−0.509	0.053
Positive	–	–	−0.394	0.146
Cognitive	–	–	−0.505	0.055
Excitement	–	–	−0.380	0.162
Negative	–	–	−0.373	0.171
	Low gamma, negative cluster			
Disorganization	–	–	−0.049	0.862
Depression	–	–	−0.177	0.529
Positive	–	–	−0.314	0.253
Cognitive	–	–	0.106	0.706
Excitement	–	–	−0.047	0.867
Negative	–	–	−0.194	0.489

**Table 4:** **Correlations between gamma-band power and performance**.

**Performance**	**Healthy controls**		**Chronic patients**	
	***r***	***p*-value**	***r***	***p*-value**
	High gamma, positive cluster			
Hits (%)	0.042		−0.056	0.836
Discrimination index A′	0.022	0.936	0.512	0.042
Reaction time (hits) (ms)	−0.125	0.646	−0.176	0.515
	Low gamma, positive cluster			
Hits (%)	0.360	0.171	−0.346	0.189
Discrimination index A′	0.454	0.077	0.404	0.120
Reaction time (hits) (ms)	0.044	0.872	−0.286	0.282
	Low gamma, negative cluster			
Hits (%)	0.300	0.260	−0.078	0.774
Discrimination index A′	0.392	0.133	−0.440	0.088
Reaction time (hits) (ms)	0.034	0.901	−0.258	0.335

### Correlations between gamma-band oscillations and medication dosage

To examine the relationship between gamma-band deficits and antipsychotic-medication, antipsychotic dose was converted to chlorpromazine equivalent levels (Woods, 2003) and correlated with gamma-band sensor power during the face condition. We found that there were no significant correlations at a corrected alpha-level of *p* = 0.016 between medication dose and high gamma-band activity over parietal channels (*r* = 0.03, *p* = 0.91). Similar results were obtained for low gamma-band activity (parietal channels: *r* = 0.48, *p* = 0.06; frontal channels: *r* = 0.29, *p* = 0.27).

## Discussion

The present study investigated the role of low and high gamma-band oscillations and the contribution of transient vs. induced activity with MEG during visual processing in chronic schizophrenia. Previous studies had reported conflicting evidence regarding the presence of deficits in gamma-band activity (Cho et al., 2006; Uhlhaas et al., 2006a; Haenschel et al., 2009). Here, we provide novel evidence for a pronounced dysfunction in high gamma-band oscillations in schizophrenia.

### Gamma-band oscillations and visual processing in schizophrenia patients

In EEG-data with the same paradigm (Uhlhaas et al., 2006a), we had reported that the amplitude of induced gamma-band oscillations in the 40–70 Hz frequency range was in the normal range while beta/gamma long-range synchronization was strongly reduced in schizophrenia. The current study gave a strikingly different result when we extended the analysis to oscillations >60 Hz. In this frequency range, we found a highly significant deficit that was extended over a large frequency range, time interval, and sensor space that has not been reported previously. This reduction in high gamma-band activity involved the transient activity related to the onset and offset of the stimulus as well as the sustained, induced response. Furthermore, the effect size of the high gamma-band impairment was pronounced (*d* = 1.23) compared to the moderate effect obtained for the lower gamma-band (*d* = 0.72). This suggests that high gamma-band oscillations may provide a more sensitive marker of impaired neural oscillations in schizophrenia that is in the range and above of effect sizes for event-related potentials that have been frequently investigated in schizophrenia, such as the P50 (de Wilde et al., 2007), P300 (Jeon and Polich, 2003), and Mismatch Match Negativity (MMN) (Umbricht and Krljes, 2005).

The deficit in high gamma-band activity showed a very robust and specific relationship with a core aspect of psychopathology in schizophrenia, namely clinical disorganization. This finding is consistent with previous psychophysical studies that demonstrated close relations between the disorganization factor and impaired perceptual organization (Uhlhaas et al., 2006b; Silverstein et al., 2000), indicating that gamma-band activity may be involved in the fragmentation of coordinated cognitive and perceptual processes as hypothesized by several theories (Phillips and Silverstein, 2003; Uhlhaas and Singer, 2010).

One reason for the difference to previous EEG-data in schizophrenia may be the difference in recording techniques. MEG provides an improved detectability of low-amplitude high-frequency oscillations with a higher SNR relative to EEG (Muthukumaraswamy and Singh, 2013). This is also reflected in the increased contribution of oscillations >60 Hz to the spectral profile that was present in the current study and has been repeatedly observed in MEG-experiments (Guggisberg et al., 2007; Chaumon et al., 2009; Grutzner et al., 2010), suggesting that MEG may be particularly suited for the investigation of high gamma-band activity during normal and abnormal brain functioning.

Interestingly, schizophrenia patients showed also stronger power over frontal and central channels in the lower gamma-band relative to controls. This change actually represented a failure to downregulate oscillatory activity in this frequency range and may be consistent with the view that frontal circuits in schizophrenia are characterized by impaired phase-resetting of stimulus related oscillatory activity (Winterer et al., 2004). From this perspective, increased low gamma-band oscillations in the current study in schizophrenia patients compared to controls could represent aberrant ongoing oscillatory activity that is normally suppressed during stimulus processing.

### Pharmacology of gamma-band oscillations

One possible mechanism for the pronounced deficits in high-frequency oscillations in schizophrenia could be impaired GABAergic neurotransmission. This hypothesis is supported by findings showing decreased functioning of parvalbumin-positive (PV) interneurons in patients with schizophrenia (Benes et al., 1997; Benes and Berretta, 2001; Lewis et al., 2012). PV-cells are of particular interest as they have been shown to underlie the generation of gamma-band activity during normal brain functioning (Sohal et al., 2009). Moreover, studies with transcranial magnetic stimulation (TMS) have demonstrated impaired cortical inhibition in schizophrenia (Daskalakis et al., 2002) which presumably reflects the integrity of inhibitory circuits because during normal brain functioning TMS-parameters of inhibition are mediated by GABA_A_ and GABA_B_ receptors (Florian et al., 2008).

More recently, *in vitro* approaches have examined the link between specific GABAergic receptor-subtypes and the generation of both high and low gamma-band activity. Oke et al. (2010) showed that GABA_A_ antagonists block the occurrence of high gamma-band activity, suggesting a close link between 60–120 Hz spectral power and GABA_A_ receptor-mediated inhibition.

In addition to GABAergic neurotransmission, glutamatergic excitatory drive has been shown to alter the generation of gamma-band oscillations. Hypofunctioning of the NDMA-receptor has been shown to dysregulate coherently organized gamma-band oscillations in distributed networks (Pinault, 2008). Moreover, selective blockade of AMPA-(alpha-amino-3-hydroxy-5-methylisoxazole-4-propionic acid-) receptors suppresses high gamma-band activity *in vitro* (Oke et al., 2010), suggesting further potential pharmacological targets for intervention in schizophrenia.

### Implications for visual dysfunctions in schizophrenia

The current findings have also implications for understanding visual dysfunctions in schizophrenia. Previous research has emphasized dorsal stream dysfunctions in visual processing in schizophrenia (Butler and Javitt, 2005). The present data provide evidence that processing in the ventral stream may also be impaired. This is supported by the behavioral deficit in detecting high contrast stimuli and recent fMRI study that demonstrated reduced activation in schizophrenia patients in higher visual cortex areas related to shape perception (Silverstein et al., 2009).

#### Non-specific task- and medication-effects on gamma-band oscillations

Overall, our results suggest that both the transient as well as the induced gamma-band response are impaired in schizophrenia, supporting the notion that perceptual dysfunctions in the present study involve impaired bottom-up as well as dysfunctions in top-down mediated activity. Possible alternative explanations for the reductions in gamma-band oscillations could involve reduced attention and abnormal eye-movements. While we cannot completely rule out these alternatives, we consider them unlikely because only correct behavioral trials were included in the analysis which minimized the contribution of reduced attention and motivation. Secondly, abnormalities in gamma-band activity have been observed under experimental conditions which do not require cognitive responses nor eye-movements, such as during ASS-paradigms (Kwon et al., 1999), suggesting that the alterations in gamma-band activity in schizophrenia represent an intrinsic feature of circuit abnormalities.

Finally, one important issue in the interpretation of deficits in gamma-band oscillations constitutes the confounding influence of anti-psychotic medication. Correlations between medication dosage and gamma-band oscillations in the current study showed no significant effect of antipsychotics on deficits in high gamma-band power. In addition, data from medication-naïve, first-episode patients with schizophrenia during the same task have revealed similar deficits (Tillmann et al., 2008), suggesting that the observed impairments in gamma-band oscillations are independent of medication status.

## Conclusion and outlook

The present study provides novel evidence for a pronounced impairment in MEG-recorded high gamma-band oscillations in schizophrenia that is accompanied by deficits in visual processing. These findings indicate impairments in local cortical networks that may underlie deficits in long-range synchronization between cortical regions as reported in previous studies (Uhlhaas et al., 2006a). Further research has to clarify to what extent deficits in local cortical circuits are directly related to functional dysconnectivity observed between brain regions or to what extent these dysfunctions may reflect independent phenomena.

In addition, links to the underlying generating mechanisms are crucial for establishing abnormalities in the gamma-band oscillations as a biomarker for translational research. Muthukumaraswamy et al. (Muthukumaraswamy et al., 2009) demonstrated that the frequency of gamma-band oscillations in MEG-recordings reflects GABA concentration. Accordingly, future research could examine the relationship between aberrant gamma-band activity and altered concentration of GABA in schizophrenia patients to establish direct links between altered physiology and brain functioning. Such research can then be further used for the identification of therapeutic strategies aimed at correcting altered neural oscillations in the disorder.

### Conflict of interest statement

The authors declare that the research was conducted in the absence of any commercial or financial relationships that could be construed as a potential conflict of interest.
